# Three new rotundabaloghid mites (Acari, Uropodina) from Sabah (Malaysia)

**DOI:** 10.3897/zookeys.447.8389

**Published:** 2014-10-16

**Authors:** Jenő Kontschán

**Affiliations:** 1 Plant Protection Institute, Centre for Agricultural Research, Hungarian Academy of Sciences, H-1525 Budapest, P.O. Box 102, Hungary

**Keywords:** East Asia, taxonomy, turtle-mites

## Abstract

Three new species of the family Rotundabaloghiidae are discovered and described from Sabah, Malaysia. The unusual *Angulobaloghia rutra* **sp. n.** differs from the other known *Angulobaloghia* Hirschmann, 1979 species in the long anterior process of the female’s genital shield. Rotundabaloghia (Circobaloghia) tobiasi **sp. n.** has very long and apically pilose dorsal setae and two pairs of bulbiform setae, which are unique in the subgenus Rotundabaloghia (Circobaloghia) Hirschmann, 1975. The long, serrate and curved setae in the big ventral cavity of Depressorotunda (Depressorotunda) serrata **sp. n.** is a so far unknown character in the subgenus Depressorotunda (Depressorotunda) Kontschán, 2010.

## Introduction

Mites of the family Rotundabaloghiidae distinctive within the Uropodina mites, having bodies that are small and rounded, ventral setae that are reduced and the marginal shield completely fused with the dorsal shield. The members of this family can be found in soil, leaf litter and moss in all tropical areas (Kontschán 2010a). Three groups of the family [the genus *Angulobaloghia* Hirschmann, 1979 and the subgenera Rotundabaloghia (Rotundabaloghia) Hirschmann, 1975 and Depressorotunda (Depressorotunda) Kontschán, 2010] are distributed only in the South-East Asian and Austral-Asian regions, one subgenus Depressorotunda (Amerorotunda) Kontschán, 2010 occurs only in South-America and the most species rich subgenus [Rotundabaloghia (Circobaloghia) Hirschmann, 1975] can be found in all tropical regions (Kontschán 2010b, 2011, Kontschán and Starý 2011, 2012). Currently four species are listed from Borneo (Kontschán 2010a) (where three countries, namely Malaysia, Indonesia and Brunie can be found), but so far no species are reported from Sabah (Malaysia). Searching the Arachnida collection of the Natural History Museum in Geneva revealed three new rotundabaloghid species in samples from Sabah, which are described in this paper.

## Material and methods

Specimens were cleared in lactic acid and drawings were made with the aid of a drawing tube. The system of nomenclature for the ventral chaetotaxy follow Kontschán’s (2010a). All specimens are stored in ethanol and deposited in the Natural History Museum in Geneva (MHNG). Abbreviations: h = hypostomal setae, St = sternal setae, im = internal malae, ad = adgenital setae, V = ventral setae. All measurements and the scales in the figures are given in micrometres (μm).

## Descriptions of new species

### Angulobaloghia rutra sp. n.

Taxon classificationAnimaliaMesostigmataRotundabaloghiidae

http://zoobank.org/187F29BC-A971-4115-AC7D-D7BF52284382

Figs 1 –10

#### Material examined.

Holotype: female. Sab-82/7. Malaysia: Sabah (Sandakan Residency): 15 milles (24 km) W de Sandakan: Sepilok: “Kabili-Sepilok Forest Reserve”, forêt près du “Pond” (étang formant la réserve d’eau pour Sepilok), tamisage de feuilles mortes et de bois pourri, Secondary Lowland Forest; 23.IV.1982; leg. B.Hauser (appareil Winkler-Moczarski à Sepilok). Paratypes: four females. Locality and date same as in holotype.

#### Diagnosis.

Genital shield of female with a long apical process and its surface covered by oval pits. Setae V7 and V8 smooth and needle-like, situated near end of pedofossae IV on small figlets. Setae on dorsal side of body pilose.

#### Description of female.

Length of idiosoma 290–320 μm, width 280–290 μm (n = 5). Shape circular, posterior margin rounded, color reddish-brown.

**Figures 1–4.**
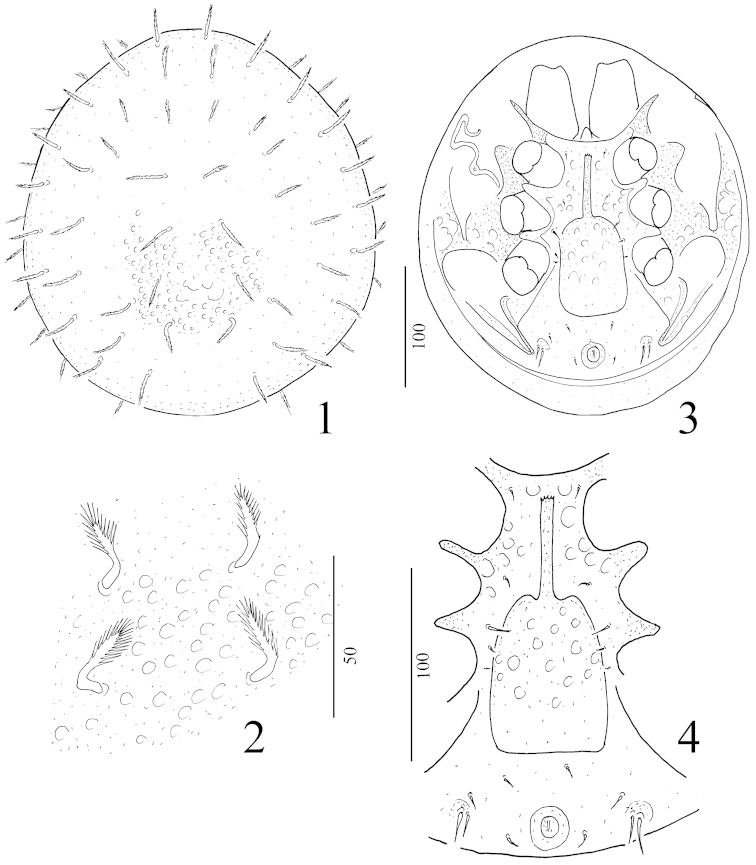
*Angulobaloghia rutra* sp. n., female, holotype: **1** body in dorsal view **2** setae and ornamentation in dorsal shield **3** body in ventral view **4** intercoxal area of female.

*Dorsal idiosoma* (Figure 1). Marginal and dorsal shields fused. Dorsal setae basally curved, margins pilose (ca 22–26 μm long) (Figure 2). Surface of dorsal body covered by small oval pits (Figure 2).

**Figures 5–10.**
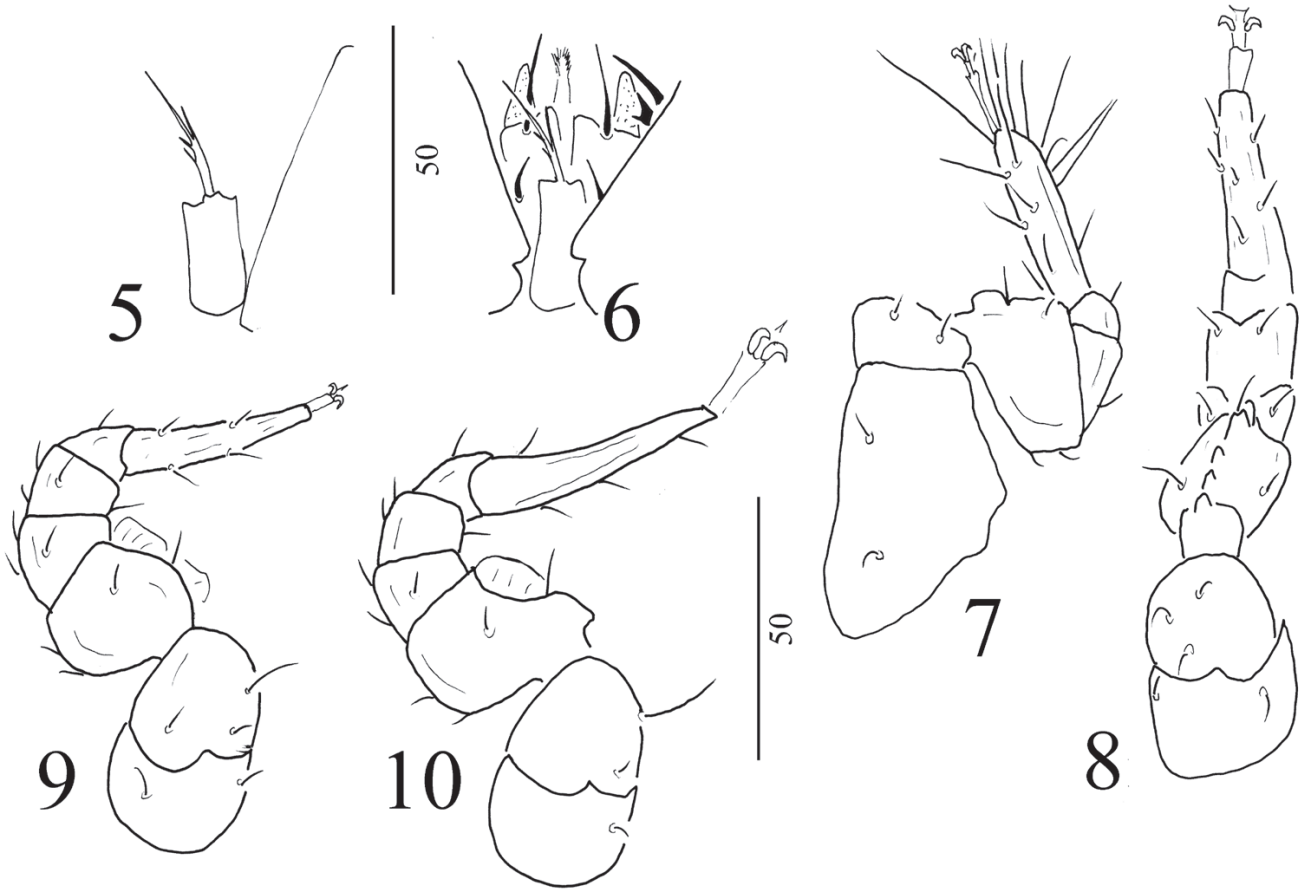
*Angulobaloghia rutra* sp. n., female, holotype: **5** tritosternum **6** ventral view of gnathosoma, tritosternum and part of coxae I **7** ventral view of leg I **8** ventral view of leg II **9** ventral view of leg III **10** ventral view of leg IV.

*Ventral idiosoma* (Figure 3). Sternal shield ornamented by oval pits (Figure 4). Sternal setae smooth, needle-like, three pairs (St1, St2 and St4) short (ca 4–5 μm) and one pair long (ca 9–10 μm). St1 situated at level of anterior margin of coxae II, St2 at level of central area of coxae II, St3 at level of posterior margin of coxae III, St4 at level of central area of coxae IV. One pair of lyriform fissures situated near St4. Ventral shield without sculptural pattern. Ventral setae smooth and needle-like. V2 (ca 5–6 μm long) situated near basal line of genital shield. V6 shorter (ca 6–7 μm), V7 (ca 10–11 μm) and V8 (ca 13–14 μm) longer and they situated near end of pedofossae IV. A small figlets bearing setae V7 and V8. Setae *ad* similar in shape and length to V2 setae, situated laterally to anal opening. Stigmata situated between coxae II and III. Peritremes hook-shaped. Genital shield linguliform with long apical process. Anterior margin of apical process serrate (Figure 4). Surface of genital shield covered by oval pits. Pedofossae deep, their surface smooth, separated furrows for tarsi IV present. Base of tritosternum narrow, vase-like, tritosternal laciniae smooth, subdivided into four smooth branches in its distal half (Figure 5).

*Gnathosoma* (Figure 6). Corniculi horn-like, internal malae smooth and short. Visible hypostomal setae as follows: h1 long (about 17–18 μm), smooth and needle-like, h2 short (about 8–9 μm), smooth and needle-like, h3 and h4 not visible (covered by coxae I). Apical part of epistome marginally pilose. Ventral side of palp trochanter with one needle-like and one robust and bifurcated setae, other setae on palp smooth and needle-like. Fixed digit of chelicerae longer than movable digit, internal sclerotized node present.

*Legs* (Figures 7–10). All legs with ambulacral claws and smooth and needle-like setae, femora II-Iv with flap-like ventral process.

Larva and nymphs, male unknown.

#### Etymology.

The name of the new species refers to the shape of the female’s genital shield. The linguliform genital shield with the long apical process resembles a shovel (= *rutrum* in Latin).

#### Remarks.

The new species differs from the other known *Angulobaloghia* species by the apical process and pit-like ornamentation of the genital shield in females. Most other known *Angulobaloghia* species have female genital shields that are triangular, semicircular or bottle-like. Only one species [*Angulobaloghia vietnamensis* (Kontschán, 2008)] has a linguliform genital shield, but the apical process is short and spine-like. In contrast, females of the new species have a long and apically serrate genial process.

### Rotundabaloghia (Circobaloghia) tobiasi sp. n.

Taxon classificationAnimaliaMesostigmataRotundabaloghiidae

http://zoobank.org/5F0276EC-A10C-4DB8-9ABD-56C039A909FD

Figs 12 –20

#### Material examined.

Holotype: female. Sab-82/15. Malaysia: Sabah (West Coast Residency): Mt Kinabalu: “Bukit Ular Trail” (sentier reliant “Kambarangan Road” à “Power Station”), tamisage de feuilles mortes et de bois pourri, forêt de *Lithocarpus*-*Castanopsis*, 1790m; 28.IV.1982; leg. B.Hauser (appareil Winkler-Moczarski à Sepilok). Paratypes: one female and one male. Locality and date same as in holotype.

#### Diagnosis.

Genital shield linguliform, its surface with irregular pits and bearing a small spine-like process on anterior margin. Setae St1 and St2 bulbiform in females. Ventral setae V2, V6, V7 and ad short, V8 four times longer than other ventral setae. Dorsal setae long and apically pilose. Surface of dorsal shield with deep and oval pits.

#### Description of female.

Length of idiosoma 370–380 μm, width 300–340 μm (n = 2). Shape circular, posterior margin rounded, color reddish brown.

**Figures 11–14.**
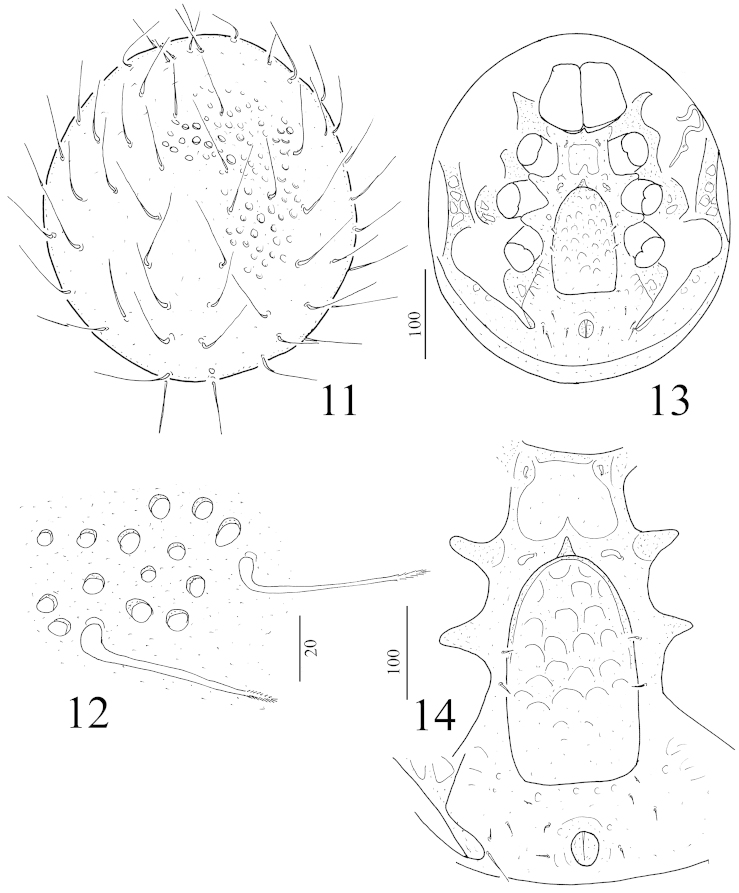
Rotundabaloghia (Circobaloghia) tobiasi sp. n., female holotype: **11** body in dorsal view **12** dorsal setae and ornamentation **13** ventral view of body **14** intercoxal area of a female paratype.

*Dorsal idiosoma* (Figure 11): Marginal and dorsal shields fused. All dorsal setae very long (ca 75–80 μm), basally curved and apically pilose (Figure 12). Dorsal idiosoma covered by deep and oval pits (Figure 12).

**Figures 15–20.**
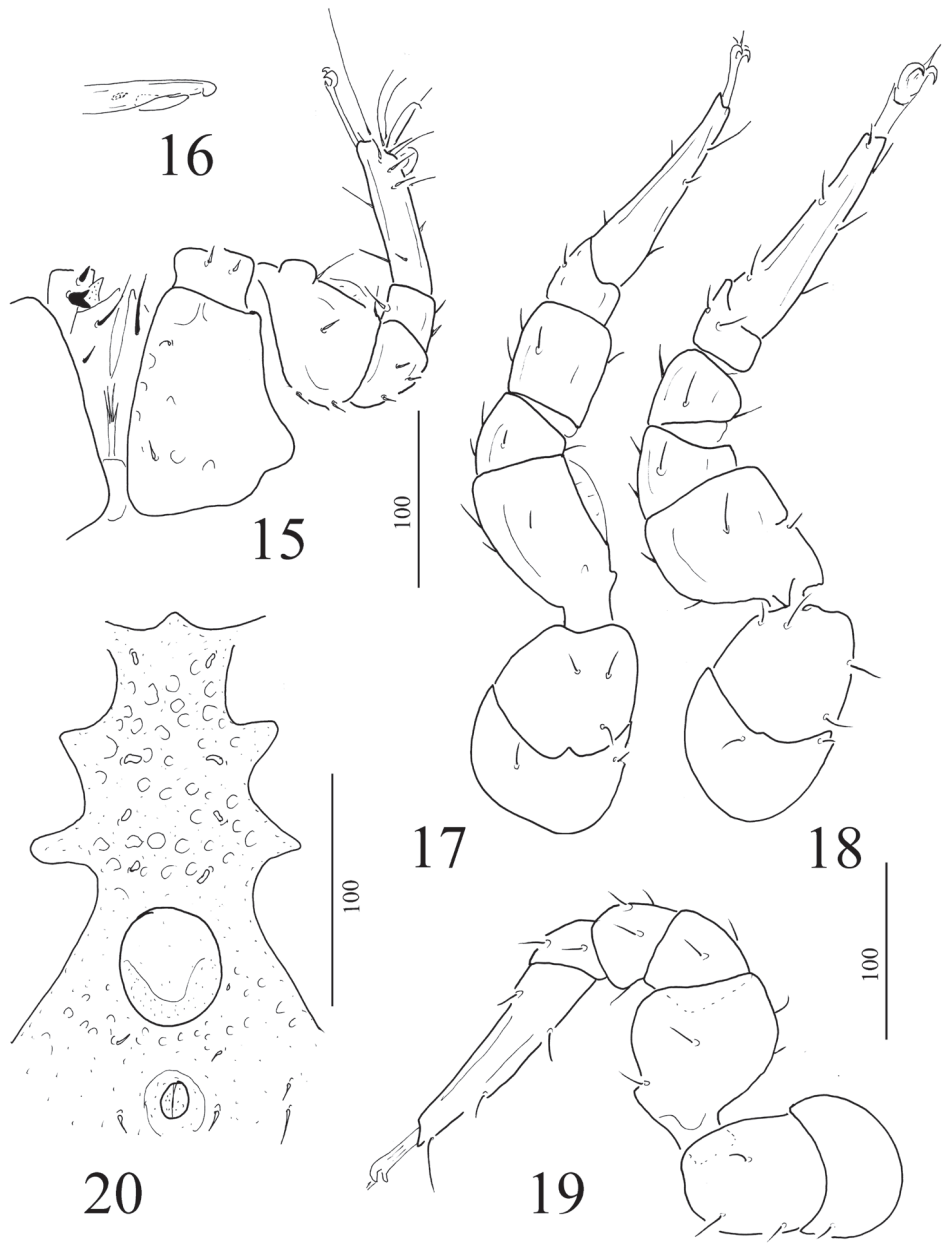
Rotundabaloghia (Circobaloghia) tobiasi sp. n., female holotype: **15** ventral view of gnathosoma, tritosternum and leg I **16** chelicera **17** ventral view of leg II **18** ventral view of leg III **19** ventral view of leg IV **20** intercoxal area of a male paratype.

*Ventral idiosoma* (Figure 13). Surface of sterna shield without sculptural pattern, only a large pit situated between coxae II. Setae St1 smooth, short (ca 4–5 μm) and bulbiform, situated near anterior margin of sternal shield, St2 smooth and bulbiform, but longer than St1 (ca 9–10 μm), situated at level of anterior margin of coxae III, St3 and St4 smooth and short (ca 6–7 μm), St3 at level of anterior margin of coxae IV, St4 at level of central area of coxae IV. St5 absent. Ventral setae smooth and needle-like. V2 (ca 7–8 μm) situated near posterior margin of genital shield (in a paratype one of V6 setae situated near to setae ad (Figure 14)). V6 short (ca 6–7 μm) and situated between V2 and V7. V7 short (ca 9–10 μm) and situated near end of pedofossae IV. V8 long (ca 15–17 μm) and situated near V7. Setae *ad* similar in shape and length to V6, lateral to anal opening. One pair of lyriform fissures placed near basal edges of genital shield. Stigmata situated between coxae II and III. Peritremes hook-shaped. Genital shield linguliform, surface with large irregular pits and its apical margin rounded and bearing a spine-like process. Pedofossae deep, their surface smooth, separated furrows for tarsi IV present. Base of tritosternum narrow, tritosternal laciniae smooth, subdivided into four smooth branches (Figure 15).

*Gnathosoma* (Figure 15). Corniculi horn-like, internal malae smooth and very short. Visible hypostomal setae as follows: h1 long (about 28–30 μm), smooth and needle-like, h2 short (about 11–13 μm), smooth and needle-like, h3 and h4 not visible (covered by coxae I). Apical part of epistome marginally pilose. Ventral side of palp trochanter with one needle-like and one robust and bifurcated setae, other setae on palp smooth and needle-like. Fixed digit of chelicerae longer than movable digit, internal sclerotized node present (Figure 16).

*Legs* (Figures 17–20). All legs with ambulacral claws and smooth and needle-like setae.

#### Description of male.

Length of idiosoma 370–410 μm, width 340–370 μm (n = 5).

*Dorsal idiosoma.* Ornamentation and chaetotaxy of dorsal shield as for female.

*Ventral idiosoma* (Figures 16). Four pairs of sternal setae bulbiform (ca. 7–8 μm) and situated anterior to genital shield. St1 situated near anterior margin of sternal shield, St2 situated at level of anterior margin of coxae III, St3 at level of posterior margin of coxae III, St 4 at level of central area of coxae IV. Surface of sternal shield with numerous oval pits. Surface of ventral shield and shape and size of ventral setae as in female. Genital shield circular and situated between coxae IV.

Larva and nymphs unknown.

#### Etymology.

I dedicate the new species to my colleague and dear friend Dr. István Tóbiás, plant virologist.

#### Remarks.

The short apical spines on the genital shield, long dorsal setae and the bulbiform sternal setae in Rotundabaloghia (Circobaloghia) tobiasi sp. n. is an unknown character combination within the subgenus Rotundabaloghia (Circobaloghia) Hirschmann, 1975.

### Depressorotunda (Depressorotunda) serrata sp. n.

Taxon classificationAnimaliaMesostigmataRotundabaloghiidae

http://zoobank.org/7F4F93C8-F905-42BF-BC68-2B1EE3A8D569

Figs 21 –31

#### Material examined.

Holotype: female. Sab-82/7. Malaysia: Sabah (Sandakan Residency): 15 milles (24 km) W de Sandakan: Sepilok: “Kabili-Sepilok Forest Reserve”, forêt près du “Pond” (étang formant la réserve d’eau pour Sepilok), tamisage de feuilles mortes et de bois pourri, Secondary Lowland Forest; 23.IV.1982; leg. B.Hauser (appareil Winkler-Moczarski à Sepilok). Paratypes: six females and three males, locality and date same as in holotype.

#### Diagnosis.

Ventral cavity oval, with one pair of long, robust, marginally serrate and curved setae. Genital shield scutiform, its surface smooth. Three pairs of short ventral setae situated near lateral margins of ventral cavity. Setae St4 very long and needle-like.

#### Description of female.

Length of idiosoma 310–330 μm, width 270–290 μm (n = 7). Shape circular, posterior margin rounded, color reddish brown.

**Figures 21–25.**
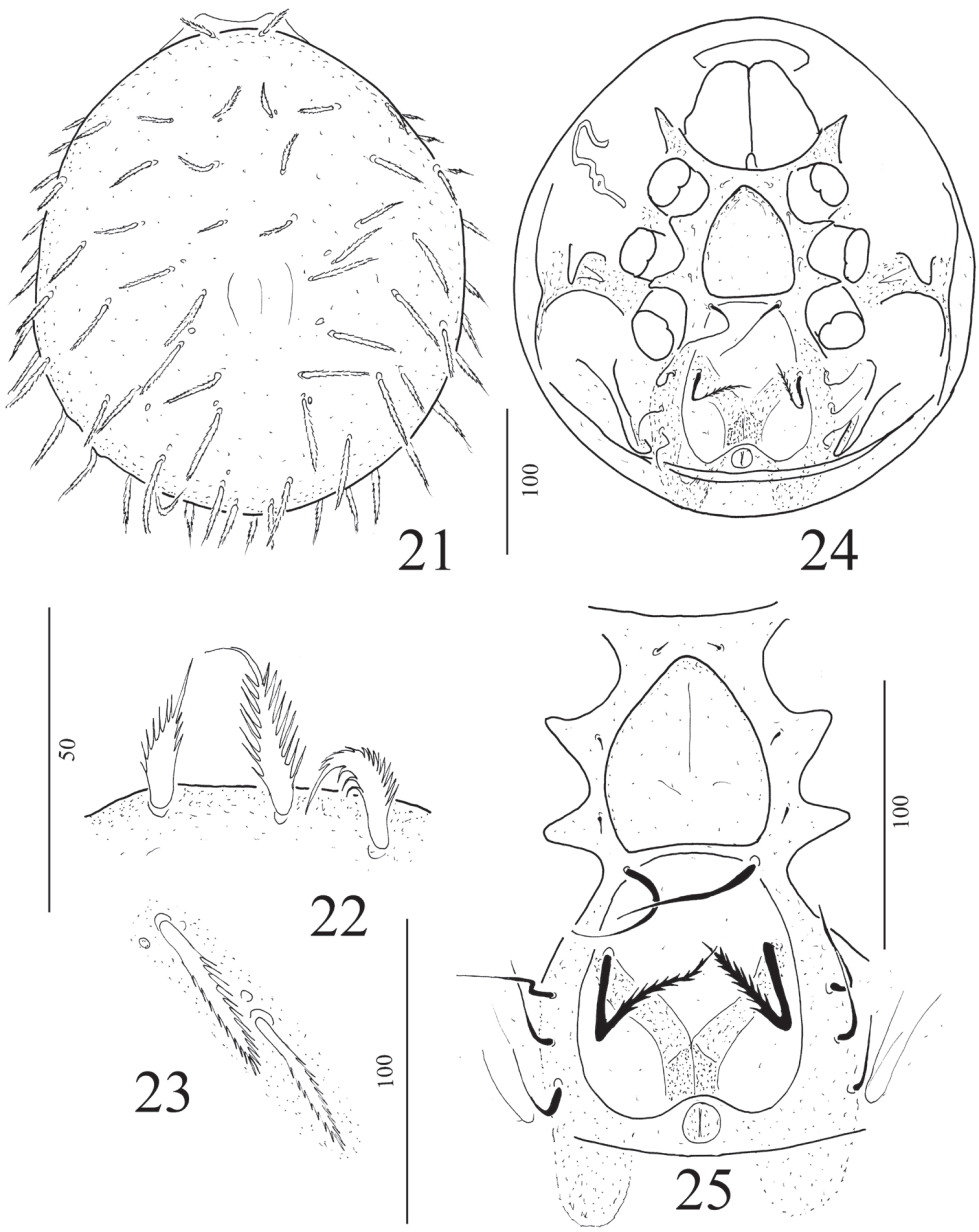
Depressorotunda (Depressorotunda) serrata sp. n. female holotype: **21** body in dorsal view **22** anterior dorsal setae **23** dorsal setae from central area of dorsal shield **14** ventral view of body **25** intercoxal area of holotype.

*Dorsal idiosoma* (Figure 21). Marginal and dorsal shields fused. Dorsal setae long (ca 42–47 μm long) and marginally pilose (Figures 23), apical setae wider than other dorsal setae (Figure 22), dorsal idiosoma without sculptural pattern.

**Figures 25–31.**
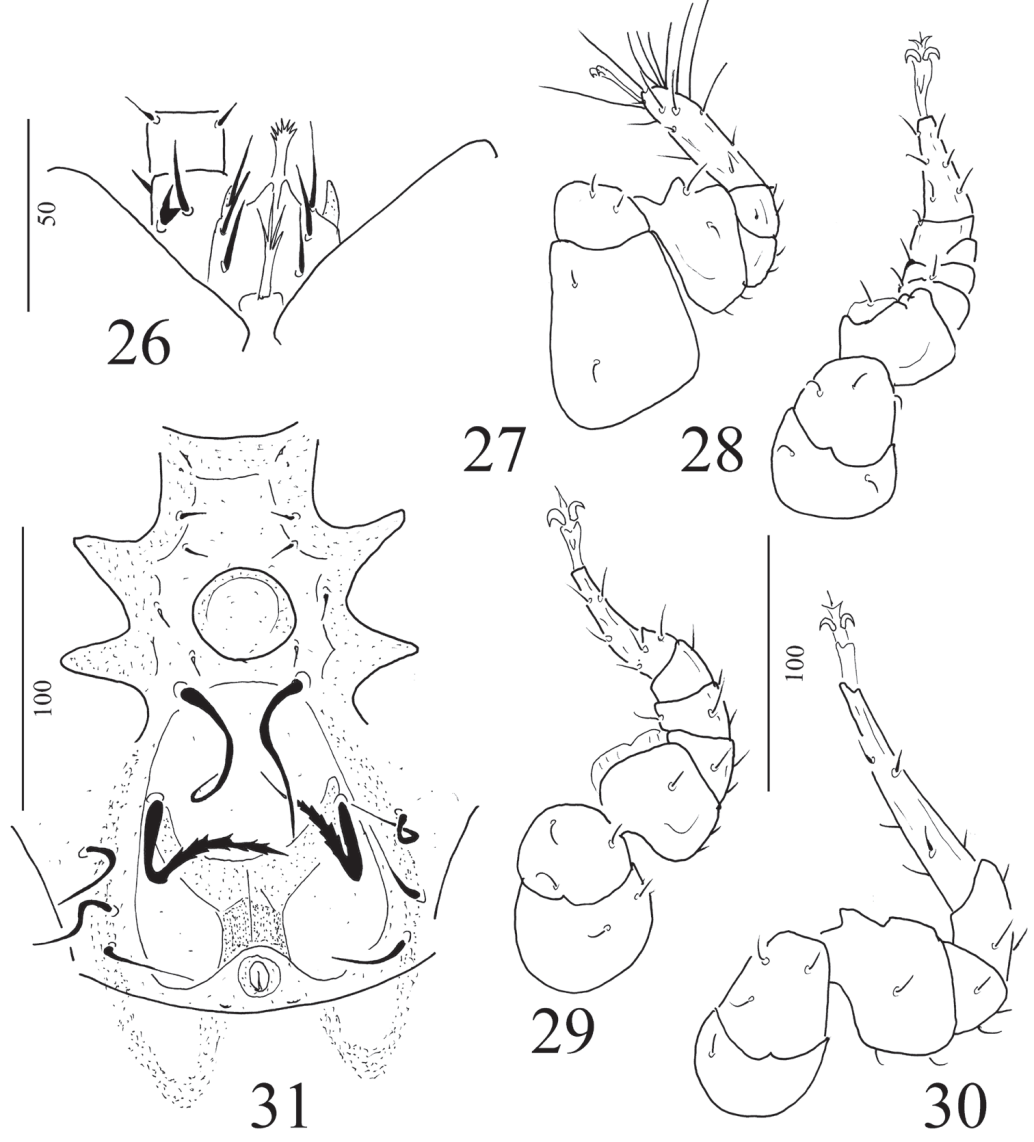
Depressorotunda (Depressorotunda) serrata sp. n. female holotype: **26** ventral view of gnathosoma, tritosternum, two segments of palp and coxae I **27** ventral view of leg I **28** ventral view of leg II **29** ventral view of leg III **30** ventral view of leg IV **31** intercoxal area of a male paratype.

*Ventral idiosoma* (Figure 24). Sternal and ventral shields without sculptural pattern. All sternal setae smooth, needle-like. Setae St1-St3 short (ca 4–6 μm), St4 robust and long (ca 58–60 μm). St1 situated at level of anterior margin of genital shield, St2 at level of posterior margin of coxae II, St3 at level of posterior margin of coxae III, St4 near basal edges of genital shield. Dorsal cavity large, oval, longer (ca 94–97 μm) than wide (ca 83–85 μm) bearing a pair of long (ca 59–62 μm) and serrate setae. Ventral setae smooth, needle-like (cat 32–35 μm) and placed in a row near margins of ventral cavity (Figure 25). Adanal setae absent. One pairs lyriform fissures situated near anterior margin of sternal shield. Stigmata situated between coxae II and III. Peritremes hook-shaped. Genital shield scutiform, surface smooth and its apical margin a little peaked. Pedofossae deep, their surface smooth, separated furrows for tarsi IV present. Base of tritosternum narrow, tritosternal laciniae smooth, subdivided into four smooth branches (Figure 26).

*Gnathosoma* (Figure 26). Corniculi horn-like, internal malae smooth and very short. Visible hypostomal setae as follows: h1, h2 and h3 long (about 18–21 μm), smooth and needle-like. Apical part of epistome marginally pilose. Ventral side of palp trochanter with one needle-like and one robust and bifurcated setae, other setae on palp smooth and needle-like. Fixed digit of chelicerae longer than movable digit, internal sclerotized node present.

*Legs* (Figures 27–30). All legs with ambulacral claws and smooth, needle-like setae.

#### Description of male.

Length of idiosoma 330–340 μm, width 300–330 μm (n = 3).

*Dorsal idiosoma*. Ornamentation and chaetotaxy of dorsal shield as for female.

*Ventral idiosoma* (Figure 31). Four pairs of short sternal setae (St1-St4) needle-like (ca. 4-6 μm), St1 and St2 situated anterior to genital shield, St3 at level of central area of genital shield and St4 at level of posterior margin of genital shield. St5 long, robust (ca. 57–59 μm) and placed near anterior margin of ventral cavity. Dorsal cavity large, oval, longer (ca 96–99 μm) than wide (ca 82–85 μm) bearing a pair of long (ca 58–62 μm) and serrate setae. One pairs lyriform fissures situated near anterior margin of sternal shield, second one pair near posterior margin of anal opening. Surface of ventral shield and shape and size of ventral setae as in female. Genital shield circular and situated between coxae III.

Larva and nymphs unknown.

#### Etymology.

The name of the new species refers to the serrate setae in ventral cavity.

#### Remarks.

The long, robust, marginally serrate and curved setae on the big ventral cavity and the extreme long St4 setae in the species Depressorotunda (Depressorotunda) serrata sp. n. are previously not observed characters within the subgenus Depressorotunda (Depressorotunda) Kontschán, 2006.

## Supplementary Material

XML Treatment for Angulobaloghia rutra

XML Treatment for Rotundabaloghia (Circobaloghia) tobiasi

XML Treatment for Depressorotunda (Depressorotunda) serrata
